# Abnormalities in visual processing amongst students with body image concerns

**DOI:** 10.5709/acp-0155-4

**Published:** 2014-05-15

**Authors:** Matthew Mundy E., Andrea Sadusky

**Affiliations:** School of Psychological Science, Monash University, Australia

**Keywords:** body image, visual processing, inversion effect, faces, bodies, scenes, body dysmorphic disorder

## Abstract

Individuals with body dysmorphic disorder (BDD) appear to possess abnormalities in the way they observe and discriminate visual information. A pre-occupation with perceived defects in appearance has been attributed to a local visual processing bias. We studied the nature of visual bias in individuals who may be at risk of developing BDD – those with high body image concerns (BICs) – by using inverted stimulus discrimination. Inversion disrupts global, configural information in favor of local, feature-based processing. 40 individuals with high BIC and 40 low BIC controls performed a discrimination task with upright and inverted faces, bodies, and scenes. Individuals with high BIC discriminated inverted faces and bodies faster than controls, and were also more accurate when discriminating inverted bodies and scenes. This reduction in inversion effect for high BIC individuals may be due to a stimulus-general local, detail-focused processing bias, which may be associated with maladaptive fixation on small features in their appearance.

## INTRODUCTION

Dissatisfaction about self-appearance is becoming more prevalent in young adults, often resulting in distress, along with social and functional impairment (Hunt, Thienhaus, & Ellwood, 2008; Spitzer, Henderson, & Zivian, 1999). Body image is generally defined as the collection of subjective emotions and beliefs which a person holds about their physical appearance and bodily functions because of the way they view themselves. *Body image* can be conceptualized as existing along a spectrum from healthy to distorted. Unhealthy (i.e., “high”) levels of *body image concern (BIC)* are typically characterized by low self-esteem, decreased social confidence, depression, anxiety, maladaptive compensatory behaviors (e.g., extreme dieting, purging, excessive grooming), or other appearance self-monitoring preoccupations (Akos & Levitt, 2002; Rosen & Ramirez, 1998). At the apex of this continuum, *body dysmorphic disorder* (BDD) is one of the many life quality-deteriorating conditions associated with (clinically significant) high BIC levels. The *Diagnostic and Statistical Manual of Mental Disorders* (DSM-IV-TR; American Psychiatric Association, 2000) defines BDD as the excessive preoccupation with one’s appearance, because there is concern about imagined or real (but minor) physical flaws. National surveys of people aged 14-99 years old estimate the prevalence of BDD to be 1.2-2.1% in the general population (Rief, Buhlmann, Wilhelm, Borkenhagen, & Brahler, 2006), with the highest rate of onset being at age 18 (Veale et al., 1996). This of particular concern, considering many individuals with BDD, eventually become housebound and unable to participate in society (Crerand & Sarwer, 2010), and some individuals will develop other DSM-IV-TR Axis I disorders alongside BDD. BDD is commonly comorbid with social phobia, misuse of drugs and alcohol, major depressive disorder, obsessive-compulsive disorder, dysthymia, eating disorders, anxiety disorders, and personality disorders (Grant & Odlaug, 2012; Gunstad & Phillips, 2003; Hunt et al., 2008). Alongside of genetic and environmental influences (Feusner, Neziroglu, Wilhelm, Mancusi, & Bohon, 2010), recent neurocognitive and behavioral research suggests that *abnormal visual processing mechanisms* are implicated in BDD; such as a bias toward bottom-up, local processing, to the detriment of a more global, gestalt, or configural representation of a stimulus (Feusner et al., 2009; Feusner, Hembacher, Moller, & Moody, 2011; Feusner, Moller, et al., 2010; Feusner, Moody, et al., 2010; Feusner, Townsend, Bystritsky, & Bookheimer, 2007). Such a bias is thought to relate to a maladaptive fixation on small details of the body or individual bodily features, which is commonly reported in BDD. This local scrutiny leads to a belief that such areas are flawed in some way, exacerbated by a lack of global bodily context.

*Global processing* (i.e., perceiving the “whole” stimulus representation) and *local processing* (i.e., perceiving a stimulus’ details) are two visual processing mechanisms which help people identify and recognize stimuli, respectively (Minnebusch & Daum, 2009). The balance between the two mechanisms is thought to be disordered in BDD. In healthy individuals, observing whether *inversion* of a stimulus (i.e., rotating it 180° from a typical exemplar orientation) affects the visual processing, discrimination, and/or recognition of that stimulus, is one method of determining which mechanism is primarily utilized for that class of stimulus. An “inversion effect” is characterized by significantly slower or less accurate visual processing of stimuli which are upside-down compared with those which are upright (Reed, Stone, Bozova, & Tanaka, 2003). Although most objects are a little more difficult to recognize in an inverted orientation compared with a “normal” orientation, inversion disproportionately disrupts the recognition of faces and bodies relative to the recognition of most other objects (e.g., scenes; Carey, 1992; Reed et al., 2003; Yin, 1969). To explain this phenomenon, inversion of a stimulus has been proposed to disrupt configural or global processing (e.g., Bruce, Doyle, Dench, & Burton, 1991; Carey, 1992; Farah, Tanaka, & Drain, 1995; Freire, Lee, & Symons, 2000). Both faces and bodies have specific configurations of features that are consistent between exemplars, which other stimuli lack. Such “second order” configuration is disrupted when a face or body is inverted (Carey, 1992; Diamond & Carey, 1986; Leder & Bruce, 2000; Reed et al., 2003). Thus, global or configural processing is thought to rely on the use of mental schemas for what stimuli classes “should” look like. For example, faces generally have two eyes above a nose and mouth, successively centered below one another (i.e., they all share second order, relational properties). Local processing, however, relies on simple, raw, feature-based visual information (Reed, Stone, Grubb, & McGoldrick, 2006). If a particular stimulus class (e.g., faces) is primarily processed using global processing, inverting such stimuli violates the second order configuration schema people have for that stimulus class (e.g., inversion causes the mouth to be above the nose and eyes - which is unlike the face composition which people are familiar with). Individuals are then forced to *switch* from global processing to local processing to identify the separate inverted stimulus features, subsequently needing to piece them together to recognize the entire stimulus. Therefore, inversion effects occur because the switch from global to local processing delays an individual’s recognition of the stimulus, and makes this interpretation more erroneous due to a lack of rehearsal in piecing upside-down features together into a cohesive whole (Reed et al., 2003, 2006).

Compared to healthy people, individuals with BDD are suspected to use a different visual processing approach which *decreases susceptibility* to inversion effects. A case-control study by Feusner, Moller, et al. (2010; see also Jefferies, Laws, & Fineberg, 2012) investigated whether individuals with BDD and healthy controls were differently impacted by inversion effects. This was achieved by successively presenting participants with a pair of face stimuli (which were both either upright or inverted), requiring participants to indicate as quickly and correctly as they could whether the faces were identical or dissimilar. In support of their hypothesis, they had found that individuals with BDD had responded significantly faster than healthy controls to inverted faces but there were no differences between the groups for upright faces. Whilst discrimination RT was impacted by inversion effects in individuals with BDD, the deterioration in their performance during inverted trials was significantly less marked than the deterioration that controls experienced. These observations implied that inversion effects were significantly diminished in BDD individuals, as compared to healthy controls. Thus, the authors proposed that their BDD group had a bias towards feature-based or bottom-up processing. That is, since the inverted faces initially required local processing to begin the recognition process, individuals with BDD appear to process both upright and inverted stimuli using predominantly local processing; their discrimination ability did not significantly differ between the two orientations. Healthy controls, however, experienced a more extensive time delay when switching from their default global processing approach (with upright faces) to a less dominant local processing (for upside-down faces). It should be noted that the performance of the healthy controls was in line with the ample evidence in the literature for the default global processing of faces (Kanwisher, McDermott, & Chun, 1997); thus, the Feusner, Moller, et al. (2010) results reveal a genuine deviation from perceptual “norms” in BDD patients.

Further support for a local processing (bottom-up) bias in BDD patients comes from functional magnetic resonance imaging studies which reported that, compared with controls, individuals with BDD showed hyperactivity in the left hemisphere (an area known for its specialized role in local processing; Gazzaniga, 2000; Love, Rouder, & Wisniewski, 1999; Proverbio, Minniti, & Zani, 1998) when viewing houses (Feusner et al., 2011) and faces (Feusner et al., 2007) regardless of their spatial resolution (i.e., whether highly detailed or with few distinguishing features). Healthy controls, however, only exhibited such activity patterns when viewing highly detailed, high resolution stimuli. This means that individuals with BDD appear to utilize the same visual processing approach regardless of the visual processing demands of a given stimulus, even when a stimulus has little detail.

Acknowledged by the authors, a weakness to the studies investigating visual processing in individuals with BDD was that they recruited patients with severe BDD, limiting the generalizability of their results to the entire BDD population (and thus perhaps those with high, pre-clinical levels of BIC). It is not currently known whether those with milder BDD, or related but non-clinical BIC, show visual processing anomalies. Thus, it is also unclear whether abnormal visual processing *precedes* and *contributes* to the development of BDD or whether it is a *result* of the disorder.

Generalizability of results to date is also weakened by a lack of direct cross-stimulus comparison. It is currently unclear whether individuals with BDD, or related BIC, primarily use local processing to recognize *faces alone* or whether this perception bias extends to other stimuli pertinent to BDD (e.g., bodies), or perhaps visual processing of all stimulus classes more generally. Feusner et al. (2011) attempted to address this issue by extending the Feusner, Moller, et al. (2010) study, by examining processing bias in house stimuli. Whilst this is an important step, the specificity of the results is somewhat clouded by using images of house stimuli which were placed within a visual “scene,” making it ambiguous whether participants were processing the house alone (an object), the scene alone (a place), or the entire image (i.e., house *within* the scene). Feusner et al. (2011) found that individuals with BDD exhibited less activity, compared with their healthy counterparts, in parahippocampal cortices (containing parahippocampal place area [PPA], known to be responsible for processing scenes; Epstein & Kanwisher, 1998). In this context, however, a reduction in PPA activity is somewhat ambiguous, since it does not differentiate between two explanations:

1. Scene processing at a global level is specifically impaired, and individuals with BDD thus show reduced activity in PPA.

2. The images are viewed more as objects than scenes, and therefore individuals with BDD attend to local features or objects within the scene, thus relying on alternative visual regions, rather than on the PPA.

Since it is known that visual processing for objects and scenes takes place in different cortical regions (e.g., Epstein & Kanwisher, 1998; Kanwisher et al., 1997; Park, Brady, Greene, & Oliva, 2011), it seems logical to separate the classes. In order to directly test whether scene perception is affected (thus representing a considerably wider visual deficit), in addition to effects seen in faces, stimuli that contain no individual object features should be tested. To further examine the breadth of the deficit, additional non-face object stimuli need to be examined, such as bodies.

Recent evidence has challenged the existence of a visual processing bias in BDD. Monzani, Krebs, Anson, Veale, and Mataix-Cols (2013) fail to see any robust differences in holistic/global versus local processing between BDD patients and controls, across several visual tasks. Significant methodological differences limit the degree to which this result can be compared with previous studies, but nevertheless indicate that more work is needed regarding the specification any contribution of visual processing abnormalities in BDD.

The present study sought to address the limitations of previous studies and assist in contributing to further elucidation of a potential stimulus-general local processing bias in BDD, along with greater generalization beyond the specific diagnosis of BDD into individuals deemed to be high on a continuum of BIC. The present methodology combined the paradigms of the Feusner, Moller, et al. (2010) BDD study and the stimulus generation protocol of Mundy, Honey, and Dwyer (2007; see also Dwyer, Mundy, Vladenau, & Honey, 2009), to investigate occurrences of inversion effects in three stimulus types (faces, bodies, and scenes), in participants with varying levels of BIC. The present study utilized images of natural landscapes (as scene sti-muli) which did not contain objects that were distinct from the rest of the scene. In order to obtain a larger, more generalizable sample, and assess potential perceptual abnormalities in individuals potentially at risk of BDD (see Feusner, Moller, et al., 2010), the present study recruited a random sample of university students who were presumed to have varying levels of BIC, in line with population norms for that demographic. Definable levels of BIC were then ascertained in this large sample via the Dysmorphic Concern Questionnaire (DCQ; Oosthuizen, Lambert, & Castle, 1998) and participants assigned to low- and high-BIC groups based on their score, before taking part in our behavioural experiment. This sample appeared suitable because the mean age of undergraduate university students fits neatly into the age range where body dissatisfaction becomes most prevalent (Veale et al., 1996). Individuals with high DCQ BIC levels are known to be at much higher risk for BDD compared to individuals with low BIC, thus these individuals may serve as pre-BDD exemplars (see Mancuso, Knoesen, & Castle, 2010). Therefore, the current study enabled the researchers to, at least partially, examine whether abnormal visual processing precedes or ensues the onset of BDD.

Using a mixed experimental design, the present study investigated whether non-clinical individuals with high BIC visually process differently to those with low BIC. Based on the theory that individuals with BDD have a bias towards local processing, it was hypothesized that participants with high BIC would demonstrate a bias towards local processing whilst individuals with low BIC would demonstrate a mix of local and global processing (to cater to the stimulus’ processing demands). Therefore, individuals with high BIC were expected to be less impacted by inversion effects than their low BIC counterparts (i.e., to show smaller differences in reaction time [RT] or accuracy between upright and inverted stimuli trials).

## Method

### Participants

Participants were recruited from Monash University, Clayton Campus, Australia, via poster advertising. Interested individuals were directed to visit a website to fill in a short questionnaire about their perception of body image. This questionnaire was an electronic version of the DCQ (Oosthuizen et al., 1998; described below). Individuals who filled in the survey received course credit.

There were 815 fully-completed online surveys. Individuals who were color blind or did not have normal or corrected (e.g., wear glasses or contacts) vision were excluded from the study. Twenty eight surveys were discarded due to aberrant responding (surveys completed in times less than two standard deviations of the mean completion time). E-mail invitations to participate in the behavioral experiment portion of the study were sent out to 51 individuals who were the lowest DCQ scorers from the sample (DCQ scores ranged from 1 to 4; 11 declined the invite) and to 49 individuals who scored the highest on the DCQ in our sample (DCQ scores ranged from 12 to 21; 9 declined the invite). In circumstances where multiple individuals from the initial DCQ cohort had identical scores (i.e., scores of 4 and 12), a random number generator was utilized to determine which student would be invited back.

The final sample consisted of 40 students (four males, 36 females; *M*_age_ = 23.40 years, *SD* = 9.14) in the low BIC group (*M*_BIC_ = 2.80, SD = 0.79), and 40 (four males, 36 females) students (*M*_age_ = 22.89 years, *SD* = 6.05) in the high BIC group (*M*_BIC_ = 17.10, *SD* = 2.73). No individuals in either group reported a clinical diagnosis of BDD. The groups were matched for sex, and there were no significant differences in age between the two groups, *t*(78) = 0.68, two-tailed, *p* = .502. To incentivize participation in the behavioral portion of the experiment, participants in the final sample were placed into a draw to win one of three gift vouchers valued at $75 each.

### Materials

The online questionnaire was hosted by *Qualtrics*©. The survey first asked a screening question regarding vision status. Individuals who reported being color blind or not having normal or well-corrected vision were directed to the end of the survey. The second portion of the survey contained questions about participants’ age, sex, and contact information. The final portion of the survey was the DCQ (Oosthuizen et al., 1998).

The DCQ (designed as a screening tool for BDD) was administered to quantify BIC. It is a seven-item self-report questionnaire, with responses on a 4-point Likert scale (from *not at all to much more than most people*). The range of possible scores is 0 to 21, with higher scores representing higher BIC levels and vice versa. Scores above 9 indicate clinical levels of BIC. The DCQ asks questions regarding survey-takers histories’ of the ways which they have perceived their body (e.g., “Have you ever: Spent a lot of time worrying about a defect in your appearance / bodily functioning?”). The questionnaire has been validated as a brief, sensitive, and specific screening instrument for BDD (Jorgensen, Castle, Roberts, & Groth-Marna, 2001; Mancuso et al., 2010; Cronbach’s alpha for the DCQ was .80). The DCQ was also shown to have a high level of convergent validity with the Body Dysmorphic Disorder Examination (BDDE; Rosen & Reiter, 1996), making this tool an ideal brief screening measure for the current study.

The face stimuli (500 × 500 pixels) were 36 pairs of color photographs originally from the MacBrain Face Stimulus Set face database (development of the MacBrain Face Stimulus Set was overseen by Nim Tottenham and supported by the John D. and Catherine T. MacArthur Foundation Research Network on Early Experience and Brain Development; Tottenham et al., 2009), and an additional stimulus set compiled by the authors. Only emotionally-neutral faces were chosen. Pairs of faces were chosen based on rough facial similarity to aid the subsequent morphing process: The software *Morpheus Photo Morpher* (ACD Systems, Saanichton, British Columbia, Canada) was then used to morph between each pair of faces so as to increase discrimination difficulty. Thus, the final 36 pairs of morphed faces, which were closer together on the morph continuum, were difficult to discriminate because they were more similar (see Mundy et al., 2007, Experiment 1, for an explanation of how the difficulty level was defined and detail on the morphing process).

Similarly, scene stimuli (500 × 500 pixels) included 36 pairs of color representations of computer generated, lifelike, virtual reality outdoor scenes (e.g., mountain ranges, coastlines) created using *Vue* 3D modeling software (E-on Software Inc, Oregon, USA). Difficult discrimination was achieved by manipulating the scenery within the 3D modeling software. An initial exemplar was created, and its partner was produced by making subtle changes in overall scenery structure, such as changes in mountain peak configuration, and morphing of the horizon (see Mundy, Downing, Dwyer, Honey, & Graham, 2013, for a more detailed description of this procedure). Such a process ensured that manipulations were made to the scenic properties of the image, and not to any individual objects.

**Figure 1.**
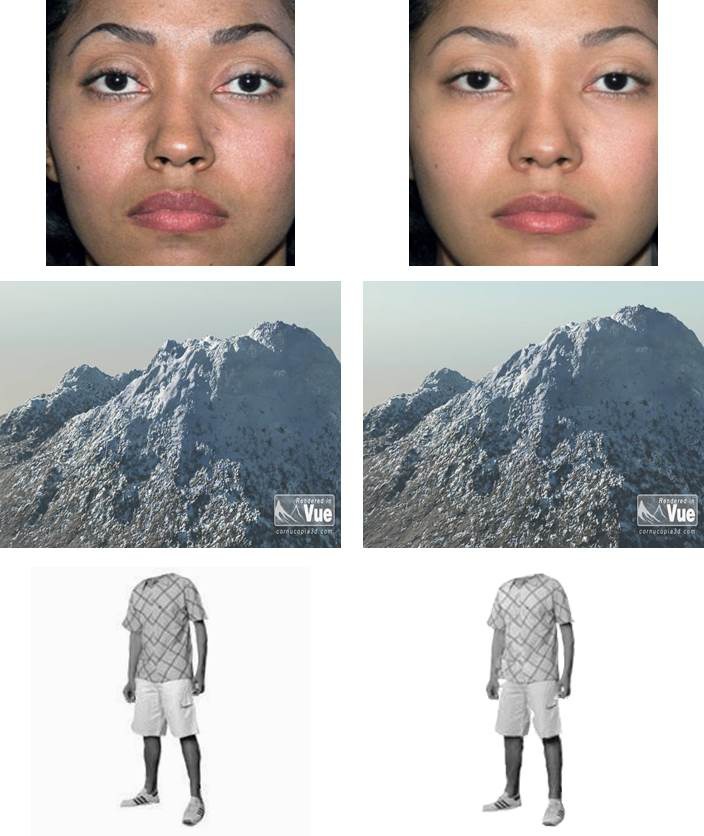
Examples of difficult to discriminate stimulus pairs used in the behavioral study.

Body stimuli (500 × 500 pixels) were created from a set of body images used by Downing, Jiang, Shuman, and Kanwisher (2001) for the purpose of activating the extrastriate body area (i.e., the region in the brain which processes the visual representation of bodies). These were clothed, full bodies without heads. Using *Morpheus Photo Morpher*, 36 pairs of bodies were morphed in an identical way to the face stimuli, to create a difficult level of discrimination. Pilot testing was conducted to ensure all selected face, scene, and body pairs were of an equivalent discrimination difficulty level. Example stimulus pairs from each category can be found in Figure 1.

An IBM compatible PC ran the behavioral experiment task. *Presentation* (Neurobehavioural Systems Inc., Albany, California, USA) was the software that was used to present stimuli and record RTs and accuracy.

### Procedure

Participants filled in the online questionnaire at their own convenience. After selection based on DCQ scores participants were called back for the behavioral experiment. Each participant was seated in a darkened room, 70 cm from a 21-in. PC computer monitor. Chair height was adjusted so that the participant’s eyes were level with the center of the computer screen. Participants were told that they would see successive pairs of stimuli appear on the screen before them and that they had to decide “as quickly and as accurately” as they could whether they believed the images were the same (by pressing the “s” key) or different (by pressing the “k” key). They were also warned about potential differences being subtle and occurring at any point during the stimulus presentation. On each trial, an initial stimulus was presented for 650 ms, followed by a blank screen for 500 ms, and then a second stimulus appeared until the participant made a same/different response. This response period was limited to 7 s before the next trial automatically began. If no response was made during this time, the trial was discarded. A black screen was then shown for 1 s before the next trial commenced.

Face, body, and scene stimuli were presented in separate blocks, with block order counterbalanced across participants. Each of the three blocks contained 288 trials, for a total of 864 trials. Within a block, each of the 36 stimulus pairs was presented 8 times, half of the stimulus pairs for a given type (18 pairs) were consistently presented upright the other half were presented in an inverted, 180° orientation. On half of the trials the two stimuli presented were the same, and on half they were different. Each block started with two practice trials, one “same” and one “different,” that contained stimuli which were not included in the experimental block. The blocks were counterbalanced across participants to ensure that half began with an inverted trial and half began with an upright trial. The entire testing session lasted approximately 60 min.

## Results

For each participant, mean discrimination accuracy and RT were calculated for each condition. Accuracy was calculated as all correct responses to same and different trials, as a percentage of the total number of trials. For RT data, we analyzed only trials for which the discrimination response was correct. SPSS Statistics 20 (International Business Machines Corporation, Australia) for Windows was utilized to run all statistical analyses. An alpha level of .05 (two-tailed) was set for all of the analyses, with multiple comparisons made via Tukey’s HSD.

### Accuracy analysis

**Figure 2.**
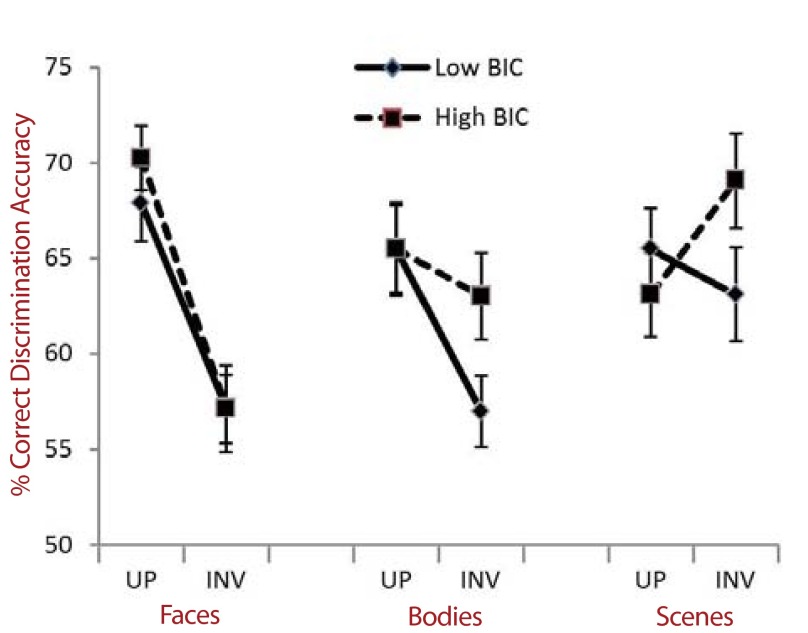
Percent discrimination accuracy data for upright (UP) and inverted (INV) face, body, and scene pairs, in both high BIC (dashed line, square markers) and low BIC (solid line, diamond markers) groups. Error bars indicate *SEM*. BIC = body image concern.

A repeated measures Stimulus (faces, scenes, bodies) × Orientation (upright, inverted) analysis of variance (ANOVA), with a between group factor of BIC Level (high, low), was conducted on the accuracy data. The interaction between Stimulus, Orientation, and BIC Level was significant, *F*(2, 156) = 13.16, *p* < .001, η^2^_partial_ = .53. This three-way interaction was qualified by two further interactions between BIC Level and Stimulus, *F*(2, 156) = 9.06, *p* < .001, η^2^_partial_ = .06; and BIC Level and Orientation, *F*(1, 78) = 27.62, *p* < .001, η^2^_partial_ = .22. These data are summarized in Figure 2.

Exploring the above interactions further, face stimuli showed a standard inversion effect, whereby participants, regardless of BIC level, were less accurate at discriminating inverted, compared with upright faces, *F*(1, 78) = 123.56, *p* < .001, η^2^_partial_ = .64. There was, however, no interaction between BIC Level and Orientation for face discrimination accuracy, *F*(1, 78) = 0.51, *p* = .822 (*ns*).

Body stimuli also showed a general inversion effect, as participants were less accurate at discriminating inverted, compared with upright bodies, *F*(1, 78) = 92.90, *p* < .001, η^2^_partial_ = .57. However, there was also an interaction between BIC Level and Orientation for body discrimination accuracy, *F*(1, 78) = 42.76, *p* < .001, η^2^_partial_ = .41. High BIC individuals were significantly more accurate at discriminating inverted bodies than low BIC individuals, *F*(1, 78) = 62.45, *p* < .001, η^2^_partial_ = .52; although the two groups did not differ in discrimination accuracy for upright bodies, *F*(1, 78) = 1.45, *p* = .230 (*ns*).

Scene stimuli showed a main effect of orientation on accuracy, *F*(1, 78) = 5.46, *p* = .022, η^2^_partial_ = .17; but also an interaction between BIC Level and Orientation, *F*(1, 78) = 5.39, *p* = .023, η^2^_partial_ = .25. High BIC individuals were significantly more accurate at discriminating inverted scenes than low BIC individuals, *F*(1, 78) = 72.22, *p* < .001, η^2^_partial_ = .37; although the two groups did not differ in discrimination accuracy for upright scenes, *F*(1, 78) = 1.15, *p* = .290. In fact, high BIC individuals displayed the opposite of a standard inversion effect whereby they were significantly more accurate at discriminating inverted scenes than upright scenes, *F*(1, 78) = 15.45, *p* < .001, η^2^_partial_ = .48.Low BIC individuals showed no inversion effect for scenes, *F*(1, 78) = 2.89, *p* = .093 (*ns*).

### Reaction time analysis

**Figure 3.**
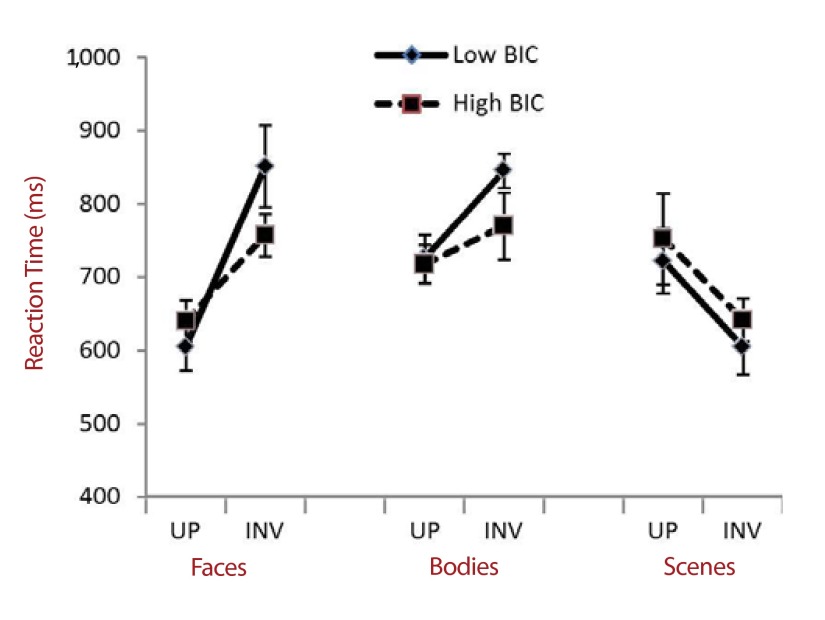
Reaction time data for upright (UP) and inverted (INV) face, body, and scene pairs, in both high BIC (dashed line, square markers) and low BIC (solid line, diamond markers) groups. Error bars indicate *SEM*. BIC = body image concern.

A repeated measures Stimulus (faces, scenes, bodies) × Orientation (upright, inverted) ANOVA, with a between group factor of BIC Level (high, low) conducted on the RT data, yielded another significant three-way interaction, *F*(2, 156) = 10.18, *p* < .001, η^2^_partial_ = .48. This interaction was qualified by two further interactions between BIC Level and Stimulus, *F*(2, 156) = 37.82, *p* < .001, η^2^_partial_ = .29; and BIC Level and orientation, *F*(1, 78) = 38.51, *p* < .001, η^2^_partial_ = .25. This data is summarized in Figure 3.

Exploring these interactions further, face stimuli showed a standard inversion effect, whereby participants were slower discriminating inverted, compared with upright faces, *F*(1, 78) = 115.92, *p* < .001, η^2^_partial_ = .55. Importantly, there was an interaction between BIC Level and Orientation for face discrimination RT, *F*(1, 78) = 12.03, *p* = .001, η^2^_partial_ = .39. High BIC individuals were significantly faster at discriminating inverted faces than low BIC individuals, *F*(1, 78) = 19.28, *p* < .001, η^2^_partial_ = .37; whereas there was no difference between the groups’ RTs to upright faces (*F* < 1, *ns*) .

Body stimuli also showed an inversion effect, as participants were slower at discriminating inverted, compared with upright bodies, *F*(1, 78) = 195.84, *p* < .001, η^2^_partial_ = .56. There was also an interaction between BIC Level and Orientation for body discrimination RT, *F*(1, 78) = 5.43, *p* = .022, η^2^_partial_ = .15. High BIC individuals were significantly faster at discriminating inverted bodies than low BIC individuals, *F*(1, 78) = 12.28, *p* < .001, η^2^_partial_ = .25; whereas there was no difference between the groups’ RTs to upright bodies (*F* < 1, *ns*) .

Scene stimuli showed a main effect of orientation on RT, *F*(1, 78) = 38.08, *p* < .001, η^2^_partial_ = .42; but in the opposite direction to the other stimulus types, whereby participants were faster to respond to inverted scenes than upright scenes. There was, however, no interaction between BIC Level and Orientation in scenes (*F* < 1).

### Correlation between DCQ scores and behavioural performance

Although the current study used dichotomous groupings of BIC based on DCQ score cutoffs, there is still value in examining a correlation between DCQ scores and behavioural performance on our tasks. If one assumes that the range of DCQ scores is analogous with a continuum of BIC, then this analysis will give some indication regarding the dimensionality of body image concerns, with regard to visual processing. Table 1 shows the correlation coefficients observed between DCQ score (participants from high and low BIC pooled together) and behavioural performance measures for each of our conditions (accuracy and RT). Inspection of the table reveals that this correlation analysis supports the interpretation of our earlier ANOVAs. Significant correlations are observed between DCQ scores and discrimination accuracy for inverted bodies and scenes (those with higher DCQ scores are more accurate than those with lower DCQ scores) as well as RTs to inverted faces and bodies (higher DCQ scores faster than lower DCQ scores). There are no other significant correlations. It should be noted, however, that any interpretation of the strength of these correlations must be limited by the dichotomous (non-continuous) nature of the sample.

**Table 1.** Pearson’s Correlation Between DCQ Score and Behavioural Performance in Each of the Test Conditions

	Faces		Bodies		Scenes	
	Upright	Inverted	Upright	Inverted	Upright	Inverted
%	.156	.098	.012	.668*	.124	.659*
RT	-.143	.743*	.119	.526*	-.097	-.056

*Note*. DCQ = the Dysmorphic Concern Questionnaire (Oosthuizen, Lambert, & Castle, 1998). % = percentage correct. RT = reaction time. *p < .01 (two tailed).

## Discussion

The present study investigated differences in visual processing mechanisms between individuals with high BIC and those with low BIC to upright and inverted stimuli. Standard inversion effects were seen for both faces and bodies in both BIC groups, in line with previous studies in healthy individuals (e.g., Carey, 1992; Reed et al., 2003; Yin, 1969). However, individuals with high BICs showed a significantly weaker inversion effect to both faces and bodies, compared with individuals who have low BICs. This difference was manifested in faster RTs for high BIC (compared to low BIC) in discriminating both inverted faces and bodies, and greater accuracy in discriminating inverted bodies (although no such difference was seen in face accuracy). Scene stimuli did not show a standard inversion effect in either group. In fact, inverted scenes were discriminated faster than upright scenes by both BIC groups. Interestingly, however, the high BIC group showed significantly greater accuracy to inverted scenes compared with the low BIC group.

Our data do not appear to be consistent with the recent findings of Monzani et al. (2013). Monzani and colleagues found no evidence for a visual processing bias in individuals with BDD. However, the tasks used by those authors and the task used here differed, particularly in terms of stimulus presentation duration, making direct comparison difficult. The current study (as well as those of Feusner and colleagues; and of Jefferies et al., 2012) used comparatively longer stimulus presentation times. It is possible, therefore, that short presentations do not afford the potential for individuals with BDD (or high BIC) to engage in the kind of processing which manifests in a local visual bias. Further testing is required to better specify the effect of exposure time on visual bias or deficit in BDD.

High BIC is one of the defining features of BDD. For the purposes of our study we have made the assumption that otherwise healthy individuals with high BIC might be considered “at risk” for BDD, although we of course acknowledge the numerous contributing factors to this condition (see also limitations below). Our findings with regard to body image and behavioral inversion effects are in direct alignment with the hypothesis and explanation of Feusner, Moller, et al. (2010): Individuals with high BIC may have a neurological activity pattern which biases them towards visually processing stimuli in a bottom-up “featural” fashion, whilst low BIC individuals are more likely to use a visual processing approach that is suitable for the stimulus at hand; which, in the case of faces and bodies, is configural or global. Like Feusner, Moller, et al. (2010), we found that individuals who score highly for BIC react more quickly to inverted faces. Building on this finding, we have also shown that high BIC individuals appear to have a similar, and potentially stronger, processing bias when discriminating bodies: High BIC individuals were both faster and more accurate than individuals with low BIC.

Such explanations sit well with the hypothesis that individuals with high BIC scrutinize (their own) appearance in a featural, piecemeal way, compared with low BIC counterparts. In this way, the self-body-dissatisfaction that individuals with high BIC have, such as those diagnosed with BDD, may be attributed to maladaptive visual processing mechanisms. Such mechanisms may skew their perception of their own body and that of others - a significant deviation from how individuals with low BIC view bodies. Feusner and colleagues suggest that individuals with BDD find features of their own and others’ appearance to be more visually salient than healthy controls. Importantly, individuals with BDD tend to report multiple concerns about their appearance (Phillips, 2005), thus have multiple regions of increased salience, which in turn, is more likely to force the break-down of a visual gestalt into separate details. In healthy participants, salient facial features have been shown to have a reduced sensitivity to the inversion effect (e.g., mouth; Barton, Keenan, & Bass, 2001). Therefore, increased salience of features in BDD and high BIC appears to disproportionality facilitate this protection from inversion.

Individuals with healthy levels of BIC are more likely to process faces and bodies holistically or globally, since these stimulus classes conform to within-group second-order configural similarity (Diamond & Carey, 1986). Presentation of inverted class exemplars is thus likely to require slower processing of features and details, due to an absence in healthy individuals of a holistic template or second-order schema for inverted faces and bodies (e.g., Freire et al., 2000). It is therefore possible that individuals with high BIC (and thus also those with clinically diagnosed BDD), who rely on a piecemeal analysis process more generally, will encode feature-based details of a stimulus faster than other individuals, regardless of orientation.

Further supporting this feature-bias contention was our finding that the high BIC group was more accurate at processing inverted scenes; and in fact inversion increased accuracy in these individuals, compared with upright orientation. Empirical support for a normal scene inversion effect in healthy participants has been mixed, with some studies that find no reductions in performance as a result of scene inversion (Wright & Roberts, 1996) or reductions that are significantly smaller than typically found in faces (Yin, 1969). Epstein, Higgins, Parker, Aguirre, and Cooperman (2006) suggest this discrepancy may be due to a difference in the way scenes and other objects are used behaviorally. For instance, scene representations are encoded to support spatial orientation and navigation within an environment, which does not necessarily require whole-stimulus matching (whereas faces are encoded in order to support exemplar identification). Thus, full configural or gestalt representations are not the only source of input when discriminating scenes. Therefore, when scenes are inverted, it may be a faster and more efficient process to switch from sensitive whole-scene representation to more robust lower-level features. If an individual already possesses a bias toward local processing, such as we suggest with high BIC, then there will be greater facilitation of this switch, resulting in greater accuracy. Regardless of the specific process at hand, our results reveal that visual processing bias associated with BDD does not appear to be limited to a single class of stimuli, nor just stimuli that are directly relevant to the disorder (faces and bodies). A stimulus-general local or featural processing bias, at the expense of global or configural processing of some stimuli, appears to be present in our high BIC group.

### Limitations

Whilst individuals with high BIC levels are known to be at much higher risk for BDD compared to individuals with low BIC (see Mancuso et al., 2010), we currently have no data to confirm whether any of our participants have subsequently been diagnosed. Therefore, it is not possible to assess whether a visual deficit precedes a clinical diagnosis. Furthermore, due to the high DCQ cutoff score for inclusion in our high BIC group, it is possible that the individuals in this group are simply *undiagnosed*, rather than pre-clinical. So whilst we can see a clear relationship between individuals without a BDD diagnosis, who are high on a continuum of BIC, and the presence of a local visual processing bias, we cannot say with certainty that this bias truly precedes the presence of BDD. We do, however, present some correlation evidence for a broader relationship between DCQ score (taken to be a measure across a continuum of BIC) and visual discrimination performance. This could be taken to indicate a more general, and thus pre-clinical, presence of processing bias in BIC. Such evidence, nevertheless, should be cautiously interpreted as it may be confounded due to the dichotomous nature of our sample, which may inflate the reported correlations. Due to limitations on the number of participants that could be tested with the behavioural paradigm, which formed part of a student project, it was not possible to test all participants that underwent the DCQ screening. Thus, a future study will focus on obtaining a cross-sectional sample containing individuals across a full range of BIC. Correlating local processing bias with BIC across this continuum will give a clearer picture of the relationship and provide stronger support to our contention that the bias precedes the presence of BDD.

### Conclusion

These results contribute to a growing body of evidence that may have clinical implications for the understanding of BDD and related disorder. The evidence presented here converges directly with previousobservations of maladaptive visual processing in disorders which display symptoms of increased BIC, such as BDD. Several studies have shown disordered face processing in such individuals (Feusner et al., 2007; Feusner, Moller, et al., 2010; Feusner, Moody, et al., 2010; Yaryura-Tobias et al., 2002), but to our knowledge, no other studies have shown an equivalent effect in body processing. As body image is a critical feature of such health issues, it would seem logical that bodies and body parts are subjected to the same level of local feature bias as faces. Importantly, this study adds a significant layer of information to our understanding of the etiology of BDD and high levels of BIC. Previously, it has not been possible to elucidate whether abnormal visual processing is an underlying trait that may *predispose* an individual to a disorder like BDD, or whether it is simply a *consequence* of the illness itself. By demonstrating a clear visual processing bias in otherwise healthy young undergraduates, predicted solely on the basis of a high level of BIC (but no clinical diagnosis of BDD), we have presented evidence which begins to support the presence of a trait predisposition towards behaviors associated with BDD. This hypothesis is further supported by our finding that local bias in visual processing is not limited to any particular class of stimuli. However, it is still unclear whether local, detailed processing biases come about as a result of *atypical neural development* or whether they are a *learned behaviour* resulting from constant attention to body features. Thus, more evidence is required before visual processing bias can be used as a cognitive marker or risk factor for BDD.

## Author Note

The authors would like to thank Julian Vilsten and Lauren Den Ouden for their help with stimulus creation and pilot testing. The authors wish to express no conflict of interest and assert that all procedures contributing to this work comply with the ethical standards of the relevant national and institutional committees on human experimentation and with the Helsinki Declaration of 1975, as revised in 2008. Funding for this study was received from the School of Psychology and Psychiatry, Monash University.
